# Multicenter, single-arm, phase II study (CAP) of radiotherapy plus liposomal irinotecan followed by camrelizumab and anti-angiogenic treatment in advanced solid tumors

**DOI:** 10.3389/fimmu.2023.1133689

**Published:** 2023-03-28

**Authors:** Jie Shen, Jing Yan, Juan Du, Xiaoqin Li, Jia Wei, Qin Liu, Hongmei Yong, Xiaolu Wang, Xiaofeng Chang, Zhou Ding, Wu Sun, Chenxi Liu, Sihui Zhu, Jingyi Guo, Huajun Li, Ying Liu, Wulou Zhang, Zonghang Liu, Rutian Li, Baorui Liu

**Affiliations:** ^1^ The Comprehensive Cancer Center of Drum Tower Hospital, Medical School of Nanjing University, Nanjing, China; ^2^ Department of Oncology, Affiliated Hospital of Jiangsu University, Zhenjiang, China; ^3^ Department of Oncology, The Affiliated Huai’an Hospital of Xuzhou Medical University and The Second People’s Hospital of Huai’an, Huai’an, China; ^4^ Department of Clinical Research and Development, Jiangsu Hengrui Pharmaceuticals Co., Ltd, Shanghai, China; ^5^ Department-II of General Surgery, Nanjing Jiangbei Hospital, Nanjing, China

**Keywords:** radiotherapy, immunotherapy, anti-angiogenic therapy, liposomal irinotecan, clinical trial, advanced solid tumors

## Abstract

**Introduction:**

Combination therapeutic mode is likely to be the key to enhance the efficacy of immunotherapy in a wider range of cancer patients. Herein, we conducted an open-label, single-arm, multicenter, phase II clinical trial that enrolled patients with advanced solid tumors who had progressed after standard treatments.

**Methods:**

Radiotherapy of 24 Gy/3 fractions/3-10 days was given to the targeted lesions. Liposomal irinotecan (80mg/m^2^, dose could be adjusted to 60 mg/m^2^ for intolerable cases) was intravenously (IV) administered once within 48 hours after radiotherapy. Then, camrelizumab (200mg IV, q3w) and anti-angiogenic drugs were given regularly until disease progression. The primary endpoint was objective response rate (ORR) in the target lesions evaluated by investigators per RECIST 1.1. The secondary endpoints were disease control rate (DCR) and treatment-related adverse events (TRAEs).

**Results:**

Between November 2020 and June 2022, 60 patients were enrolled. The median follow-up was 9.0 months (95% confidence interval (CI) 5.5-12.5). Of 52 evaluable patients, the overall ORR and DCR were 34.6% and 82.7%, respectively. Fifty patients with target lesions were evaluable, the ORR and DCR of the target lesions were 35.3% and 82.4%, respectively. The median progression-free survival was 5.3 months (95% CI 3.6, 6.2), and the median overall survival was not reached. TRAEs (all grades) occurred in 55 (91.7%) patients. The most common grade 3-4 TRAEs were lymphopenia (31.7%), anemia (10.0%), and leukopenia (10.0%).

**Conclusion:**

The combination of radiotherapy, liposomal irinotecan, camrelizumab, and anti-angiogenesis therapy demonstrated promising anti-tumor activity and well tolerance in various advanced solid tumors.

**Clinical trial registration:**

https://clinicaltrials.gov/ct2/home, identifier NCT04569916.

## Introduction

Although immunotherapy has become a powerful weapon for a wide variety of cancer types, its benefit in the overall population is extremely low. Developing effective immune-combination therapy is a potential strategy to benefit a wider range of patients, especially those who have failed standard therapies.

Stereotactic body radiotherapy (SBRT) is a classical and effective locoregional therapy for cancer. In addition to directly causing DNA damage in tumor cells, it also stimulates tumor-specific antigens release, thereby enhancing the antitumor immune response (1). Previous clinical studies have demonstrated that radiotherapy combined with PD-1/PD-L1 inhibitors has long-lasting antitumor activity in advanced or metastatic melanoma (2), non-small cell lung cancer (NSCLC) (3), Hodgkin lymphoma (HL), renal cell carcinoma (RCC) and other cancer types (4).

Nowadays, nanotechnology combined with RT and immunotherapy arises a novel pathway for boosting cancer therapy effect. Liposomal irinotecan is a liposomal formulation of irinotecan, which is one of the well-known chemotherapeutic agents with radiosensitizing property. Liposomes have protective effect on encapsulated drugs and can specifically deliver drugs to tumors owing to enhanced permeability and retention effect (5). Previous study shows that irinotecan plus SBRT have promising results in metastatic colorectal cancer, despite heavily pretreated patients (6). Nanoparticles (NPs) distribution and accumulation were up-regulated by the interaction between RT and tumor microenvironment (TME) (7). Thereby we proposed that SBRT combined with liposomal irinotecan may systematically ameliorate tumor immunity and increase tumor control.

Moreover, preclinical and clinical studies have shown that radiotherapy has a synergistic effect with immunotherapy *via* a stimulating adaptive immune response (8), and positively regulating the TME (9) to promote the anti-tumor response. The combination therapy has been proved to be effective and safe in the treatment of varieties of tumors (10–12). Furthermore, crosstalk between tumor vessels and immune cells determines the nature of anti-tumor immunity, which contributes to a destructive cycle in tumor growth (13). Emerging preclinical evidence demonstrates the potential of anti-angiogenesis therapy could improve the efficacy of cancer immunotherapy by reprogramming the immune microenvironment *via* vessel normalization (14, 15). Treatment of camrelizumab (an anti-PD-1 antibody) and apatinib (an anti-angiogenic agent) alone or in combination has been proved to have antitumor activity in nasopharyngeal carcinoma (NPC), head and neck cancer (HNC), hepatocarcinoma (HCC), esophageal squamous cell carcinoma and other types of cancers (16–18).

Based on the abovementioned interactions and synergy, a multi-modal approach combining radiotherapy with liposomal irinotecan, anti-angiogenic therapy, and immunotherapy is a promising therapeutic strategy. To our best knowledge, no clinical trials have investigated this pattern of combination treatment. Herein, we present our phase II study of SBRT with liposomal irinotecan, camrelizumab, and anti-angiogenic therapy for patients with advanced solid tumors who have failed or have no available standard therapy or lack of systemic therapy.

## Patients and methods

### Study design and patients

This was a single-arm, multicenter, open-label, phase II study (NCT04569916). The main center of the clinical trial was Nanjing Drum Tower Hospital affiliated to Nanjing University Medical School, and sub-centers were Affiliated Hospital of Jiangsu University and Huai’an Second People’s Hospital. This study was approved by the ethics committees of the above three hospitals. Patients provided written informed consent.

Eligible patients were aged 18 years or older, pathologically confirmed with recurrence or metastasis solid tumors (such as pancreatic cancer, colorectal cancer, NSCLC, HCC, HNC, gastric cancer, etc.). Patients have progressed on or lacked systemic therapy. Other key inclusion criteria included at least two measurable lesions (at least one extracranial measurable lesion for patients with brain metastases) according to Response Evaluation Criteria in Solid Tumors version 1.1 (RECIST 1.1), an Eastern Cooperative Oncology Group (ECOG) performance score of 0-2, a life expectancy of at least 12 weeks and had adequate organ function. The complete list of eligibility criteria is provided in **Supplementary Material 1**. All patients provided written informed consent before study participation. The trial was carried out in accordance with the International Conference on Good Clinical Practice Standards and the Declaration of Helsinki.

### Procedures

Eligible patients received hypofractionated radiotherapy after enrollment (a total irradiation dose of 15-24Gy in 3 fractions, delivered at 5-8Gy per fraction, completed within 3-10 days). Liposomal irinotecan (80 mg/m^2^, ivdrip) was given once within 48 hours after the last radiotherapy. Dose could be adjusted to 60 mg/m^2^ for intolerable cases judged by investigator. Camrelizumab (200mg, ivdrip, within 30-60 min) was administrated within 48 hours after the treatment of liposomal irinotecan in 21-day cycles, combined with anti-angiogenic therapy. Apatinib 250mg qd was the first choice of anti-angiogenic drug. If patients were intolerant of anti-angiogenic drug, drug withdrawal, interruption, and modification were permitted.

### Outcomes

The primary endpoint was the objective response rate (ORR) of the target lesions. Radiographic tumor response evaluation was performed using CT obtained after camrelizumab every 2 cycles until disease progression or unacceptable toxic effects. Tumor response was determined based on RECIST 1.1.

The secondary endpoints included disease control rate (DCR) of target lesions, treatment-related adverse events (TRAEs), and clinical benefit rate (CBR). TRAEs and serious adverse events (SAEs) were collected and evaluated according to the National Cancer Institute Common Terminology Criteria for Adverse Events (NCI CTCAE) (version 4.0.3).

### Statistical analysis

The full analysis set (FAS) was defined as all patients enrolled and received at least one dose of any drug in study treatment, which was the primary efficacy analysis set. The safety analysis set included all patients who received at least one dose of any study drug and recorded safety data after administration. Trial results are mainly analyzed using descriptive statistics. Overall survival (OS), median progression-free survival (mPFS), and duration of response (DoR) were estimated using the Kaplan-Meier method, and the 95% CI was calculated using Brookmeyer and Crowley methods. In addition, survival plots are plotted. The chi-square test was used to assess the associations between prior treatment and clinical outcomes. Statistical significance was defined as *P* < 0.05.

## Results

### Patient population and baseline characteristics

**Figure 1:**
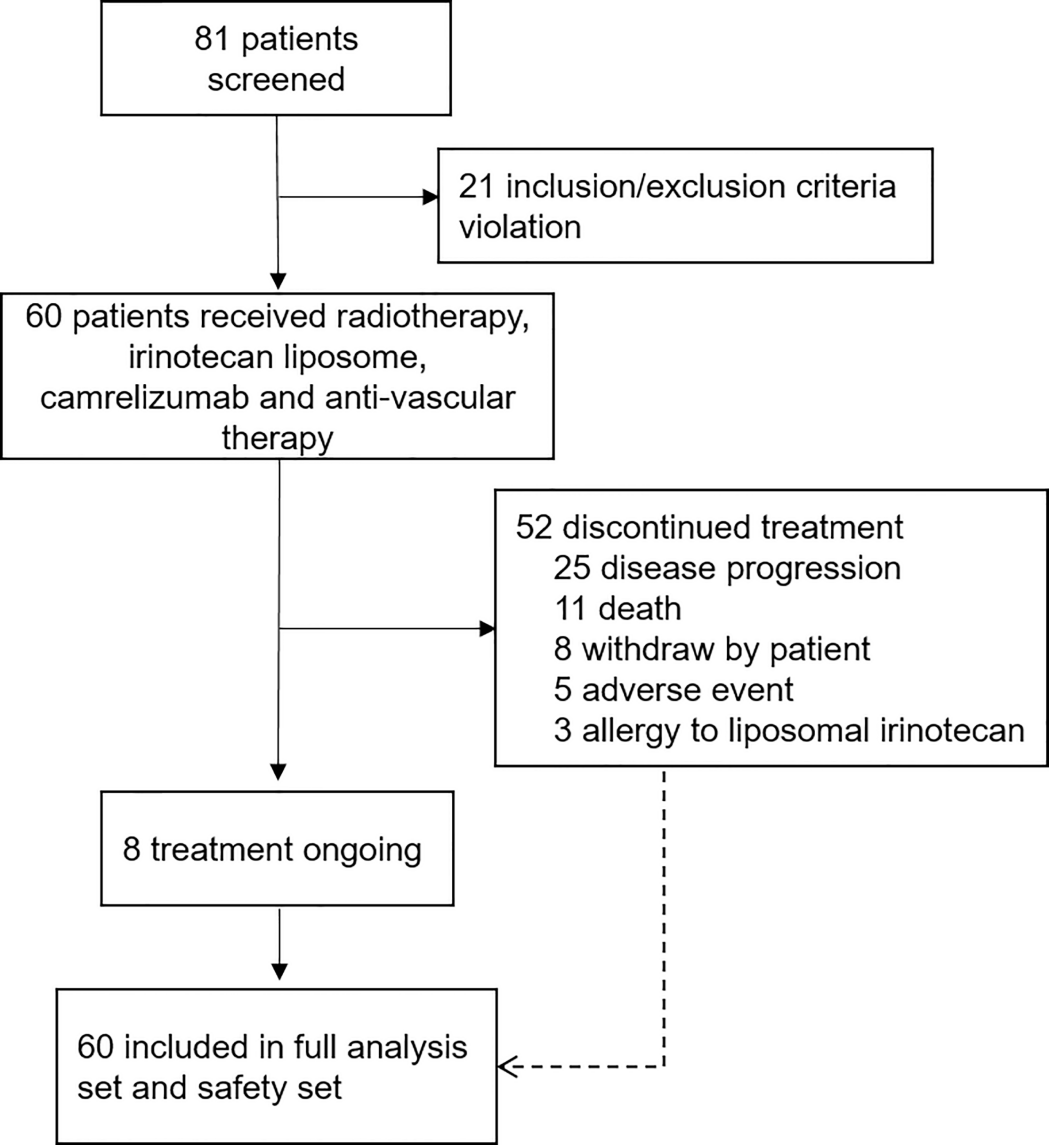
The flowchart of enrolled patients.

Between November 2020, and June 2022, a total of 60 eligible patients were enrolled (**Figure 1**). All patients were evaluable for toxicity, and 50 were radiographically evaluable for efficacy.

The demographic and disease characteristics at baseline are listed in **Table 1**. The median age was 58 years (range, 21-84 years), 33 patients (55.0%) were male and 27 (45.0%) were female. Of these, 10 patients (16.7%) did not receive systemic therapy before enrollment due to lack of standard treatment or intolerance of chemotherapy, and 28 (46.7%) received third- or later-line therapy. 20 (33.3%), 17 (28.3%) and 21 (35.0%) patients received prior radiotherapy, immunotherapy or anti-angiogenic drugs, respectively. Main reasons for discontinuing study treatment were disease progression (PD) (41.7%, 25/60) and death (18.3%, 11/60). Thirty-eight patients (63.3%) received apatinib, 3 (5.0%) received anlotinib, 4 (6.7%) received lenvatinib, and 15 (25.0%) did not receive any anti-angiogenic drugs during the study due to poor performance status.

**Table 1:** Patient baseline demographics and disease characteristics.

Characteristic	N (%)
**Median age,years (range)**	58 (21-84)
**Sex**	
Male	33 (55.0%)
Female	27 (45.0%)
**ECOG score**	
0	17 (28.3%)
1	31 (51.7%)
2	12 (20.0%)
**No. of prior anticancer therapies**	
0	10 (16.7%)
1	12 (20.0%)
2	10 (16.7%)
≥3	28 (46.7%)
**Prior therapy**	
Radiotherapy	20 (33.3%)
Anti-PD1/PD-L1	17 (28.3%)
Anti-VEGF	21 (35.0%)
**No. of metastatic sites**	
0-2	39 (65.0%)
≥3	21 (35.0%)

### Efficacy

At the time of data cutoff (June, 2022), the median duration of follow-up was 9.0 months (95% CI 5.5-12.5). A total of 52 patients were evaluable by RECIST 1.1 criteria, while other 8 patients were not evaluable owing to death or falling off. In terms of overall efficacy, 18 patients had partial response (PR), 24 had stable disease (SD) and 9 had progressive disease (PD). The overall ORR and DCR were 34.6% and 82.7%, respectively (**Supplementary Table S1**).

We particularly analyzed the efficacy of patients with target lesions. Of 52 evaluable patients, 50 patients with target lesions were evaluable by RECIST 1.1 criteria, while target lesions of two were unevaluable. The ORR and DCR of patients with target lesions were 36.0% and 96.0%, respectively, as PR was observed in 18 patients, SD in 30 patients, and PD in 2 patients (**Supplementary Table S1**). Among patients with target lesions, we also found that objective response occurred in more patients without prior anti-angiogenic therapy than those with prior treatment (ORR: 45.2% *vs.* 21.1%, *P*=0.085). In addition, patients who were not pre-treated with immunotherapy seem more likely to benefit from the treatment with an ORR of 48.5%, compared with those with prior immunotherapy (ORR: 11.8%, *P*=0.01). The complete list is provided in **Supplementary Table S2**.

**Figure 2:**
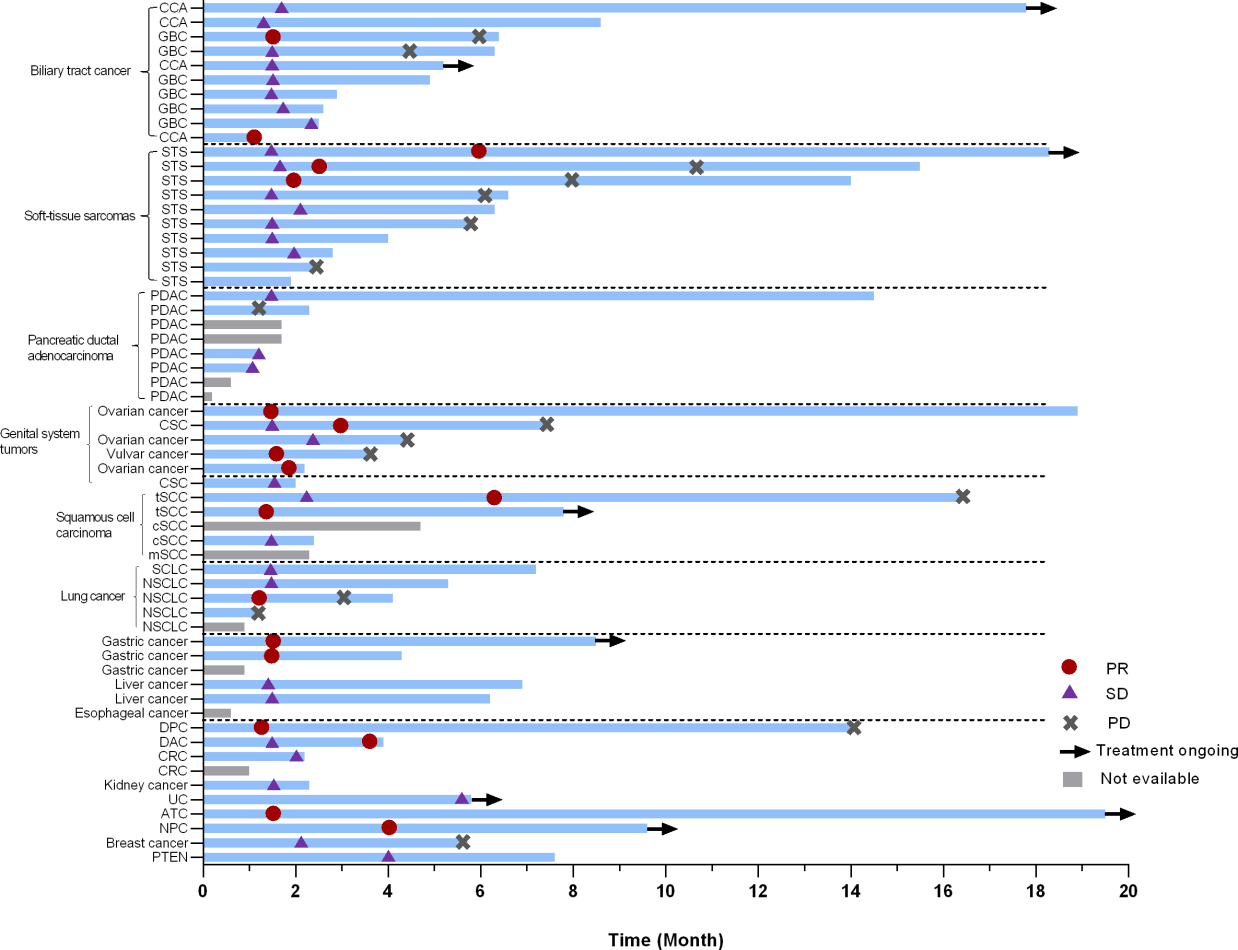
Swimmer plot according to cancer type. PR, partial response; SD, stable disease; PD, progressive disease; CCA, cholangiocarcinoma; GBC, gallbladder carcinoma; STS, soft-tissue sarcomas; SCLC, small lung cancer; NSCLC, non-small lung cancer; PDAC, pancreatic ductal adenocarcinoma; CSC, cutaneous squamous cell carcinoma; tSCC, thymic squamous cell carcinoma; cSCC, cutaneous squamous cell carcinoma; mSCC, metastatic squamous cell carcinoma of unknown primary;DPC, duodenal papilla carcinoma; DAC, duodenal adenocarcinoma; CRC, colorectal cancer; UC, Urothelial carcinoma; ATC, Anaplastic thyroid carcinoma; NPC, nasopharyngeal carcinoma; PNET, pancreatic Neuroendocrine Tumor.

In the current study, more than 20 types of cancer were included. Nearly half of the subjects were with digestive system tumors including 10 with biliary tract cancer (2 PR, 8 SD), 8 with pancreatic cancer (4 evaluable, 4 SD), 3 with gastric cancer (GC) (2 evaluable, 2 PR), and 2 with duodenal carcinoma (2 PR). Additionally, ten patients with soft-tissue sarcomas (STS) were enrolled, six of which were undifferentiated sarcomas. The ORR was 30.0% (3/10) in STS. Besides some common cancer types, such as ovarian cancer (2 PR, 1 SD) and non-small cell lung cancer (3 evaluable, 1 PR, 1 SD, 1 PD), a few of rare types of cancer were also under investigation in our study. For instance, one patient was with anaplastic thyroid cancer (ATC) (PR), and two were with thymic squamous cell carcinoma (2 PR). The best response of target lesions of each cancer type is shown in **Table 2** and the swimmer plot according to cancer type was shown in **Figure 2**.

**Table 2:** Overall efficiency and the target lesions evaluation.

	Evaluable patients	PR	SD	PD	ORR	DCR
Efficiency of the targetlesions	50	18	30	2	36.0%	96.0%
Overall efficiency	52	18	25	9	34.6%	82.7%

**Figure 3:**
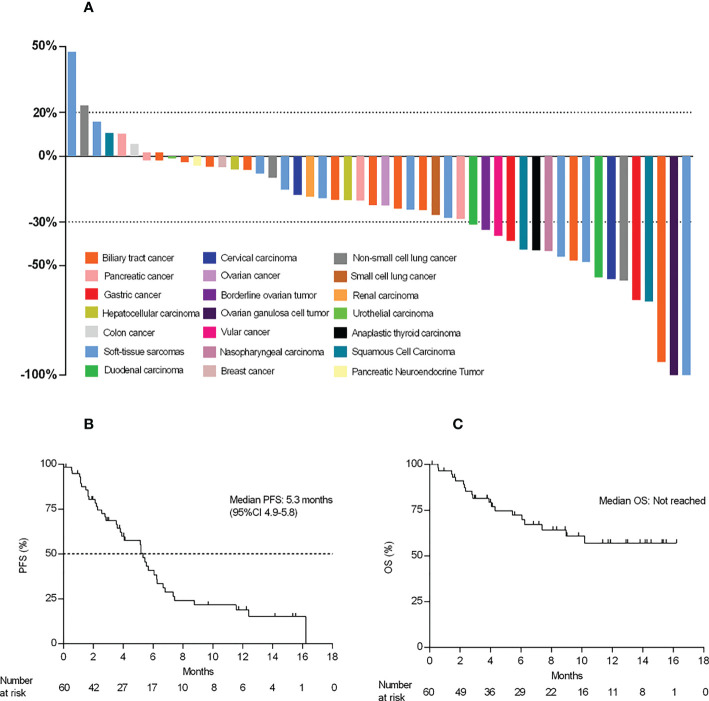
Waterfall plot and survival data. **(A)** Best percent change from baseline in the target lesions (n=50). **(B, C)**, Kaplan–Meier curve of PFS and OS.

As shown in **Figure 3A**, a total of 42 patients achieved a shrinkage of their target lesions. Among 18 patients who had PR, the median time to response (TTR) was 2.3 months (95%CI 1.2, 3.4). The median duration of response was 7.5 (95%CI 3.0, NA), and the CBR was 30.0%.

At the time of data cutoff, eight patients (13.3%) were still receiving treatment, 40 (66.7%) patients had PFS events, and the mPFS was 5.3 months (95% CI 3.6, 6.2) (**Figures 3B, C**). A total of 19 patients (31.7%) died, and the mOS was not reached (NR) (95% CI 7.4, NA) (**Figures 3B, C**). The estimated one-year overall survival rate was 57.1% (95%CI 0.4-0.8).

### Safety

All of the 60 patients were included in the safety analysis. The median cycle of camrelizumab was 4 cycles (range, 4–28). A total of 55 (91.7%) of the 60 patients experienced at least one TRAE (**Tables 3**, **4**). The most common any-grade TRAEs were lymphocyte count decreased (56.7%), anemia (43.3%), white blood cell count decreased (41.7%), rash (28.3%), and hypokalemia (28.3%). Grade 3 or 4 TRAEs occurred in 45.0% (27/60) of patients. The most common AEs were lymphocyte count decreased (31.7%), anemia (10.0%), and white blood cell count decreased (10.0%). Treatment related SAEs of grade 3 or higher were reported in 3 patients (5.0%), including neutrophil count decreased (2 patients, 3.3%) and diarrhea (1 patient, 1.7%).

**Table 3:** Efficacy of each cancer types.

Cancer Types	Number of patients enrolled	Number of evaluable	The best efficacy of target lesions (N)
**Digestive System Tumors**			
Biliary tract cancer	10	10	PR (2), SD (8)
Pancreatic cancer	8	4	SD (4)
Gastric cancer	3	2	PR (2)
Hepatocellular carcinoma	2	2	SD (2)
Duodenal carcinoma	2	2	PR (2)
Colon cancer	2	1	SD
Esophageal cancer	1	0	NE
**Soft-tissue sarcomas**			
Undifferentiated sarcomas	6	6	PR (2), SD (4)
Synovial sarcoma	2	2	SD (1), PD (1)
Myxofibrosarcoma	1	1	SD
Fibrosarcoma	1	1	PR
**Genital System Tumors**			
Cervical carcinoma	2	2	PR (1), SD (1)
Ovarian cancer	1	1	SD (1)
Borderline ovarian tumor	1	1	PR (1)
Ovarian granulosa celltumor	1	1	PR (1)
Vulvar cancer	1	1	PR (1)
**Respiratory System Tumors**			
Non-small cell lung cancer	4	3	PR (1), SD (1), PD (1)
Small cell lung cancer	1	1	SD
**Squamous Cell Carcinoma**				
Cutaneous squamous cell carcinoma	2	1	SD (1)
Thymic squamous cell carcinoma	2	2	PR (2)
Metastatic squamous cell carcinoma of unknown primary	1	0	NE
**Urinary System Tumours**				
Urothelial carcinoma	1	1	PD (1)
Renal carcinoma	1	1	SD
**Endocrine System Tumour**			
Anaplastic thyroid carcinoma	1	1	PR (1)
**Head and Neck Cancer**				
Nasopharyngeal carcinoma	1	1	PR (1)
**Breast Cancer**	1	1	SD (1)
**Pancreatic Neuroendocrine Tumor**	1	1	SD (1)

NE; Not available.

**Table 4:** Treatment related adverse events.

TRAE	Grade 1-4		Grade 3-4	
Total	55	91.7%	27	45.0%
Lymphocyte count decreased	34	56.7%	19	31.7%
Anaemia	26	43.3%	6	10.0%
White blood cell count decreased	25	41.7%	6	10.0%
Hypokalaemia	17	28.3%		
Rash	17	28.3%		
Alanine aminotransferase increased	16	26.7%	2	3.3%
Diarrhoea	13	21.7%	1	1.7%
Platelet count decreased	13	21.7%	1	1.7%
Aspartate aminotransferase increased	12	20.0%		
Neutrophil count decreased	11	18.3%	3	5.0%
Hypoalbuminaemia	9	15.0%		
Pyrexia	9	15.0%		
Asthenia	9	15.0%	1	1.7%
Vomiting	8	13.3%		
Haematuria	6	10.0%		
Nausea	5	8.3%		
Abdominal pain	5	8.3%		
Bilirubin conjugated increased	5	8.3%	1	1.7%
Hypersensitivity	3	5.0%	3	5.0%
Protein urine present	3	5.0%		
Glomerular filtration rate decreased	3	5.0%		
Pain	3	5.0%		
Alopecia	3	5.0%		
Chest discomfort	3	5.0%		
Blood bilirubin increased	3	5.0%		
Blood creatinine increased	3	5.0%	1	1.7%
Proteinuria	2	3.3%		
Hypoproteinaemia	2	3.3%		
Hyperuricaemia	2	3.3%		
Cough	2	3.3%		
Blood urine present	2	3.3%		
Occult blood positive	2	3.3%		
Decreased appetite	2	3.3%		
Gastrointestinal disorder	2	3.3%	2	3.3%
Blood bilirubin decreased	2	3.3%		

TRAEs leading to camrelizumab treatment interruption occurred in 7 patients (11.7%). Patients discontinued camrelizumab because of TRAEs that were lymphopenia and leukopenia. TRAEs leading to apatinib treatment interruption occurred in 10 patients (16.7%). Only three patients discontinued liposomal irinetocan owing to hypersensitivity reactions during infusion.

Immune-related AEs (irAEs) of any grade occurred in 47 (78.3%) patients, and the most frequent irAEs were lymphocyte count decreased (56.7%), white blood cell count decreased (31.7%), anemia (30.0%), and rash (26.7%).

## Discussion

In this phase II study, we attempted to improve the antitumor efficacy *via* a combination treatment with radiotherapy, liposomal irinotecan, camrelizumab and anti-angiogenic drugs in patients with advanced solid tumors who have failed or have no available standard therapy or lack of systemic therapy.

Given that patients in our study generally had no standard of care, or progressed on multi-line therapy in the advanced-stage of tumor, good tolerance and low toxicities were the major concerning. A clinical trial has shown that hypofractionated radiation therapy (3 fractions of 8 Gy) combined with anti-PD-L1-antibody or anti-CTLA-4 antibody could induce the proliferation of CD8^+^ T-cell and increase of M1/M2 macrophage ratios in patients with metastatic microsatellite stable colorectal adenocarcinoma (19). Considering the poor performance status and therapeutic tolerance of the enrolled patients, we adopted 3 fractions of 5-8 Gy, which is not only conducive to activating the body’s anti-tumor immune response, but also well tolerated by the patients.

We found the comprehensive therapeutic strategy provided a promising anti-tumor activity for advanced patients with tolerable toxicities, regardless they received prior immuno- or anti-angiogenic therapy or not. Especially, several types of tumors demonstrated promising efficacy and well-tolerate safety that deserve further exploration.

### Soft-tissue sarcoma

Sarcomas are a heterogenous group of cancers can be further divided based on distinct morphology and genetic changes into more than 100 subtypes as defined by the World Health Organization (20). Due to the multiple subtypes, high heterogeneity, and low immunogenicity of STSs, the pathological types of the patients enrolled in previous sarcoma studies were different, and the efficacy was not ideal. Patients with STS progressing after anthracycline-based chemotherapy, naïve from angiogenesis inhibitors received anlotinib as the second-line drug, the efficacy was with an ORR of 12% and mPFS of 5.6 months, of undifferentiated sarcomas, mPFS was 4.1 months and mOS was 11 months (21). A pooled analysis of phase II trials investigating a PD1 or PD-L1 antagonist in patients with advanced STS, the ORR of PD-1/PD-L1 antibody as single-agent immunotherapy was 15.8% (22). In terms of immunotherapy combined with TKI, pembrolizumab combined with axitinib had an ORR of 25%, and TQB2450 (PD-L1 antibody) combined with anlotinib display a good performance with an ORR of 36.7%, mPFS of 7.9 months (23). In CAP study, ten patients with STS were enrolled, including six undifferentiated sarcomas. The ORR in STS was 33.0% (3/10), mPFS was 6.2 months (95%CI:1.9-10.5), mOS was NR. Notably, two of six undifferentiated sarcomas got PR, which showed that undifferentiated sarcoma was relatively easy to achieve remission. The comprehensive treatment regime of immunotherapy combined with anti-angiogenic therapy, radiotherapy, and chemotherapy deserved further exploration in the treatment of undifferentiated sarcoma.

### Gastric cancer

Three patients with GC were enrolled in this study. Two patients could be evaluated for efficacy and were still in follow-up, of which the best efficacy was PR, and PFS was not reached. The response rate of this study in GC was higher than that of immunotherapy combined anti-angiogenic therapy or immunotherapy combined chemotherapy (24). Survival data was not reached, and the sample size needed to be expanded to verify.

### Duodenal carcinoma

Duodenal carcinoma (DA) is a rare tumor, accounting for less than 1% of gastrointestinal malignancies. The median survival of the patients who underwent resectional operation was 54 months (25). Practical guidelines for the treatment of DA are lacking and there is limited data reporting outcomes for metastatic duodenal adenocarcinoma (26). The gene mutation spectrum of small intestinal adenocarcinoma, colorectal cancer and GC is very different, and the molecular characteristics of unspecified small intestinal adenocarcinoma and duodenal adenocarcinoma are also different (27). Two patients with duodenal carcinoma were enrolled in this research, and the efficacy was PR. mPFS of the patients were 16.2 months and 3.8 months, respectively. In the future, we will explore efficacy correlation in genomic changes and TME of DA.

### Anaplastic thyroid cancer

Anaplastic thyroid cancers (ATCs) comprise 1% to 10% of all thyroid cancers worldwide. Patients with ATCs have a median survival of 5 to 12 months and a 1-year overall survival of 20% to 40% (28). One patient with ATC was enrolled in this research. The best efficacy was PR and PFS exceeded 15.3 months. The patient was still in follow-up. Dabrafenib combined with trametinib have been FDA-approved for the treatment of ATC patients with BRAF V600E mutation, while patients without BRAF V600E mutation have no systemic therapy (29). This research provides a comprehensive treatment regime for patients with wild-type BRAF V600E ATC without systemic therapy, so that patients can obtain long-term remission.

We recognize the limitations of our study. This study enrolled a heterogeneous patient population, with a limited number of patients in a variety of primary cancers. Moreover, patients were exposed to a high number of prior therapies, and their physical capability and compliance are relatively poor, thus some patients did not use angiogenesis drugs because of poor performance status, intolerance, financial reasons, or other subjective causes. Furthermore, the immune status of these patients such as PD-L1 expression before and during therapy was not prespecified in our protocol, and whether PD-L1 expression could be consider as a biomarker for the efficacy of the study combination regimen in this challenging disease these warranted further investigation in future trials with larger sample size. Exploring effective predictive biomarkers would be our future work since it is vital to identify which cancer types and populations are particularly suitable for combinatorial approaches.

## Conclusion

Regardless of whether the patients previously had received radiotherapy, immunotherapy or anti-angiogenic drugs, the patients progressing on multi-line therapy could still benefit from radiotherapy in combination with liposomal irinotecan, camrelizumab and anti-angiogenic. For rare tumors without prior systemic therapy (such as certain sarcomas, duodenal carcinoma, and ansplastic thyroid carcinoma), the efficacy of this therapy was better than the existing therapies.

## Data availability statement

The original contributions presented in the study are included in the article/**Supplementary Material**. Further inquiries can be directed to the corresponding authors.

## Ethics statement

The studies involving human participants were reviewed and approved by the ethics committees of Nanjing Drum Tower Hospital, Affiliated Hospital of Jiangsu University and Huai’an Second People’s Hospital. The patients/participants provided their written informed consent to participate in this study.

## Author contributions

JS: Methodology, writing-original draft, patient enrollment, treatment, project administration, investigation, data curation, formal analysis, writing-review and editing. JY: Patient enrollment, treatment. JD: Patient enrollment, treatment. XL: Patient enrollment, treatment. JW: Patient enrollment, treatment. QL: Patient enrollment, treatment. HY: Patient enrollment, treatment. XW: Patient enrollment, treatment. XC: Patient enrollment, treatment. ZD: Patient enrollment, treatment. WS: Patient enrollment, treatment. CL: Methodology, writing-review and editing, specimen collection, data entry. SZ: Specimen collection, data entry. JG: Specimen collection, data entry. HL: Resources. YL: Resources. WZ: specimen collection. ZL: specimen collection. RL: Conceptualization, methodology, investigation, project administration. BL: Conceptualization, methodology, investigation, project administration, writing-original draft, patient enrollment, treatment. All authors contributed to the article and approved the submitted version.
